# Terpenes and Cannabinoids in Supercritical CO_2_ Extracts of Industrial Hemp Inflorescences: Optimization of Extraction, Antiradical and Antibacterial Activity

**DOI:** 10.3390/ph15091117

**Published:** 2022-09-07

**Authors:** Stela Jokić, Igor Jerković, Valentina Pavić, Krunoslav Aladić, Maja Molnar, Martina Jakovljević Kovač, Sanda Vladimir-Knežević

**Affiliations:** 1 Faculty of Food Technology, Josip Juraj Strossmayer University of Osijek, Franje Kuhača 18, 31000 Osijek, Croatia; 2 Faculty of Chemistry and Technology, University of Split, Ruđera Boškovića 35, 21000 Split, Croatia; 3 Department of Biology, Josip Juraj Strossmayer University of Osijek, Cara Hadrijana 8/A, 31000 Osijek, Croatia; 4 Faculty of Pharmacy and Biochemistry, University of Zagreb, A. Kovačića 1, 10000 Zagreb, Croatia

**Keywords:** *Cannabis sativa*, industrial hemp, supercritical CO_2_ extraction, terpenes, cannabinoids, antiradical activity, antibacterial activity

## Abstract

Natural products are increasingly in demand in dermatology and cosmetology. In the present study, highly valuable supercritical CO_2_ (sCO_2_) extracts rich in bioactive compounds with antiradical and antibacterial activity were obtained from the inflorescences of industrial hemp. Volatile compounds were analyzed by gas chromatography in tandem with mass spectrometry (GC-MS), while cannabinoids were determined by high performance liquid chromatography (HPLC-DAD). Extraction yields varied from 0.75 to 8.83%, depending on the pressure and temperature applied. The extract obtained at 320 bar and 40 °C with the highest content (305.8 µg mg^−1^) of cannabidiolic acid (CBDA) showed the best antiradical properties. All tested extract concentrations from 10.42 µg mL^−1^ to 66.03 µg mL^−1^ possessed inhibitory activities against *E. coli*, *P. aeruginosa, B. subtilis*, and *S. aureus*. The sCO_2_ extract with the highest content of cannabidiol (CBD) and rich in α-pinene, β-pinene, β-myrcene, and limonene was the most effective. The optimal conditions for sCO_2_ extraction of cannabinoids and volatile terpenes from industrial hemp were determined. The temperature of 60 °C proved to be optimal for all responses studied, while the pressure showed a different effect depending on the compounds targeted. A low pressure of 131.2 bar was optimal for the extraction of monoterpenes, while extracts rich in sesquiterpenes were obtained at 319.7 bar. A high pressure of 284.78 bar was optimal for the extraction of CBD.

## 1. Introduction

Currently, there is a growing demand for products made from *Cannabis sativa* L., a medicinal plant that has experienced significant controversy throughout history. The official approval of the medicinal use of cannabis and cannabinoids worldwide has stimulated research in the field of dermatology and cosmetology. Although the topical application of cannabinoids is still in its infancy, there is obviously a growing interest in such preparations. Science has yet to thoroughly research the efficacy of cannabinoids and the safety of their application to the skin, but the production and use of dermatological and cosmetic preparations is increasing daily [1]. A recent study on the prevalence and causes of topical cannabis use in the Canadian population was published by Mahmood et al. [2]. Topical cannabis preparations were used at least once by 24.3% of respondents for the most common dermatologic conditions, such as atopic dermatitis (25%), acne (19%) and anti-ageing skin care (16%) and for non-dermatologic conditions such as joint stiffness (30%) and headaches and migraines (27%). Creams (26%) were the most common form of hemp-based preparations [2].

The main constituents of this plant species are cannabinoids and terpenes, which are mainly contained in the inflorescences. Unlike medicinal cannabis, industrial hemp contains less than 0.3% of the psychoactive cannabinoid tetrahydrocannabinol (THC), while other non-psychoactive cannabinoids are present in much higher proportions [3]. Terpenes also play an important role, as they are volatile components of the plant that have been shown to have synergistic effects with cannabinoids [4]. Nowadays, industrial hemp is a promising renewable resource that can be used not only for the production of textiles, paper, or biofuels, but also in the food, pharmaceutical, and cosmetic industries [5]. Therefore, the production of high quality extracts with a defined composition of desired bioactive compounds is of increasing importance. Today’s research is focused on finding environmentally friendly methods for the extraction, isolation, and purification of cannabinoids, as well as on the development of methods for their qualitative and quantitative analysis.

In the extraction of plant material, the requirements of modern production are almost completely met by supercritical CO_2_ (sCO_2_) extraction, as it is one of the “clean technologies” that does not produce environmentally harmful by-products. The sCO_2_ extraction is based on the fact that CO_2_ near the critical point or in the supercritical range is a very efficient solvent for certain types of compounds. The fluid is in a supercritical state at a temperature above its critical temperature (*T*_c_) and a pressure above its critical pressure (*p*_c_). In this range, the density of the fluid approaches that of liquids, the viscosity approaches that of gases, and diffusion is higher than for conventional solvents. By adjusting the process parameters of pressure and temperature during extraction, the selectivity of the fluids for chemically very sensitive phytochemicals can be influenced [6,7]. Thus, this extraction technique is highly selective and also “green” and is therefore selected in this study for the extraction of cannabinoids and terpenes from industrial hemp. The sCO_2_ extraction is increasingly used for the isolation of cannabinoids and terpenes from medicinal and industrial hemp. Previous research has focused on the optimization of extraction conditions, solubility of individual cannabinoids in sCO_2_, their fractionation, and evaluation of the extraction efficiency compared to other extraction techniques [8]. Perrotin-Brunel et al. investigated the solubility of non-psychoactive cannabinoids in sCO_2_ compared to THC to separate them from the mixture. Lower temperatures were required for good solubility of cannabidiol (CBD) and cannabinol (CBN) than for cannabigerol (CBG) and tetrahydrocannabinol (THC), which dissolve well only at high temperatures, which may be related to differences in chemical structure and melting points [9]. It was found that, in addition to optimizing pressure and temperature, the use of ethanol as a co-solvent significantly contributes to extraction efficiency, shortens extraction time, and reduces the use of solvent and plant material to achieve high yields. Pulsatile addition of co-solvents showed better results than constant concentration of co-solvents [10]. Gallo-Molina et al. optimized the process of extraction, isolation, and purification of THC using supercritical fluid extraction and solid phase extraction [11]. Extracts rich in CBD and related cannabidiolic acid (CBDA) were obtained from biomass residues after industrial hemp processing using an optimized extraction procedure with sCO_2_ and fractionation [12]. A specially developed method for the supercritical extraction of eleven cannabinoids from medicinal hemp and purification with special filters and additional separation chambers was also described [13]. 

The sCO_2_ extracts from industrial hemp, rich in terpenes and cannabinoids without psychoactive effects, have great potential for application in the pharmaceutical and cosmetic industries. The beneficial effects of cannabinoids on the skin and the possibility of their therapeutic action for various skin diseases, such as eczema, acne, itchy skin, systemic sclerosis, and alopecia, have been studied [1,14]. Among cannabinoids, CBD, already included in many topical preparations, stands out for its antioxidant, anti-inflammatory, and analgesic effects, as well as for its positive effect on wrinkle reduction and skin hydration. Although the potential of CBD has been recognized, future clinical trials are expected to provide sufficient evidence for its efficacy and safety [14,15]. Modern methods of CBD extraction, isolation, and purification, including sCO_2_ extraction, have been researched and developed [16].

The objectives of this research were: (a) to determine the optimal conditions for the isolation of the desired phytochemicals (terpenes and cannabinoids) from the inflorescences of industrial hemp by applying “green” extraction with CO_2_ under supercritical conditions; (b) to identify the terpenes in the obtained sCO_2_ extracts by GC-MS; (c) to quantify the content of cannabinoids in the obtained extracts by HPLC; (d) to determine the antioxidant and antimicrobial activities of the sCO_2_ extracts. As far as we are aware, no one has yet provided detailed insight into the full composition of the volatile terpenes and cannabinoids of the sCO_2_ extracts from industrial hemp in terms of biological activity, which is the focus of the present research. The complete phytochemical analysis of the extracts from industrial hemp and the analysis of their antioxidant and antibacterial properties as well as the optimization of the extraction may help to ensure their inclusion in new cosmetic products, which is highly desirable. 

## 2. Results and Discussion

### 2.1. Supercritical CO_2_ Extraction of Industrial Hemp

Research on sCO_2_ extraction of bioactive compounds from industrial hemp inflorescences is accessible only through the few published papers, and the demand for such natural extracts is increasing. As detailed in the introduction, most of these studies are related to the solubility of selected cannabinoids or to residues of industrial hemp and medicinal cannabis; only a few hemp compounds have been studied. It is known that sCO_2_ extraction is a highly selective technique and that varying the pressure at constant temperature changes the solvent density [9], which may affect the solubility of components, such as terpenes and cannabinoids. In order to optimize the extraction, the influence of the two main process parameters of sCO_2_ extraction (pressure and temperature) on the yield and the content of bioactive extract constituents was investigated. The experimental matrix was prepared according to the Central Composite Design (CCD) (Table 1), and the analysis of variance (ANOVA) evaluated the degree of accuracy of the applied methodology. Extraction yield is calculated as the total mass of extract obtained (g) divided by the mass (g) of plant material placed in the extractor and expressed as a percentage (%).

Table 1 shows that the extraction yield varied from 0.76 (run 6) to 8.83% (run 8). This was expected due to the application of different extraction pressures ranging from 78.6 bar to 361.4 bar. The lower limit of 78.6 bar was only slightly above the critical CO_2_ solvent pressure of 74 bar, and it is known that volatiles are better extracted at lower pressures. However, higher pressures resulted in much higher yields, so an optimization process is required. In addition, the selected upper temperature limit (64.1 °C) was low enough to avoid changes in thermolabile compounds. 

**Figure 1:**
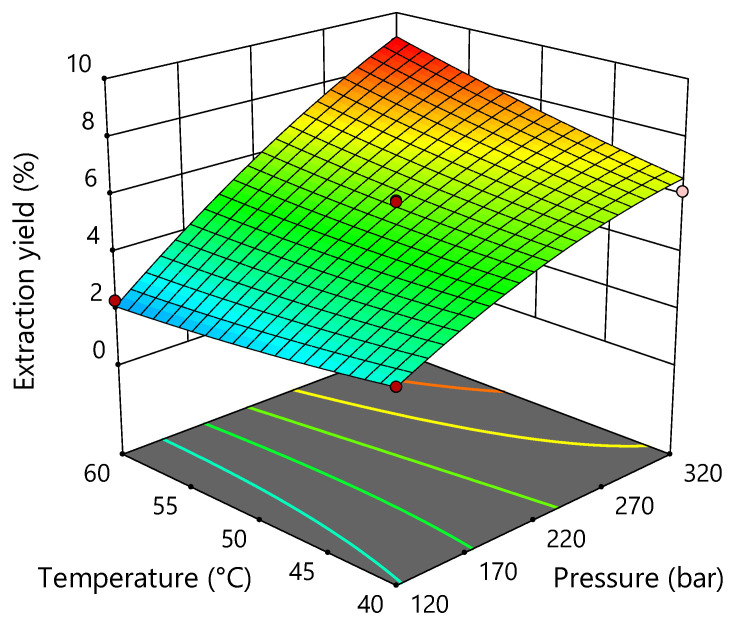
Three-dimensional plot for obtained extraction yield as a function of extraction pressure and temperature.

Based on the results of ANOVA, listed in the Supplementary Materials (Table S1), the regression model for yield was highly significant (*p* < 0.0001), and the obtained coefficient of determination R^2^ value was 0.9868 with a non-significant lack of fit (*p* = 0.0557), showing a reasonable representation between the input parameters and the observed extraction yield. In addition, pressure was the most statistically significant parameter for the observed response (*p* < 0.0001), indicating the expected increase in yield at higher pressure (Figure 1). From Table S1, it can be seen that temperature had a statistically strong influence on yield, but it was not as significant as pressure. In addition, quadratic terms of pressure and temperature variables showed a significant effect on yield, while the interaction between extraction parameters had no influence (*p* = 0.3578).

Very similar results for extraction yield were also obtained in a previous study [17], where the yield ranged from 0.33 to 7.13% for industrial hemp variety Helena, extracted with sCO_2_. The extraction yield increased with increasing pressure at constant temperature and ranged from 1.57 to 5.20% at 40 °C, from 0.78 to 5.71 % at 50 °C, and from 0.33 to 7.13% at 60 °C. In several studies investigating the effects of pressure on the yield of supercritical extraction, an increase in yield with increasing pressure was observed in the extraction of oil from industrial hemp seeds [18], as well as cannabinoids from industrial hemp consisting of leaves, flower fragments, and immature seeds [19], and cannabinoids from flowers of different hemp varieties [10]. Kitrytė et al. investigated the effects of pressure (100–500 bar) and temperature (35–70 °C) on the extraction yield from hemp threshing residues. Optimum conditions were found to be 46.5 MPa and 70 °C, resulting in a yield of 8.3%. The response surface diagram for the yield also showed a very similar shape to that in this study. However, it is important to note that the extraction yield depends on the part of the plant to be extracted and also on the plant itself, i.e., plant variety, location, and growing conditions [10]. 

### 2.2. Terpenes in sCO_2_ Extracts of Industrial Hemp

The sCO_2_ proved to be a good solvent for the extraction of volatile compounds, such as terpenes from *C. sativa* [20]. Mono- and sesquiterpenes are largely responsible for the characteristic aroma of *C. sativa*. Their biosynthesis has been studied [21], and terpene synthases from *C. sativa* have been characterized [22,23]. They were identified ubiquitously in the essential oil of this plant [24]. Recently, comprehensive methods for the isolation of terpene compounds from *C. sativa* have been presented [20], including hydrodistillation (HD), conventional solvent extraction (SE), and sCO_2_ extraction. Proven and innovative extraction protocols and chromatographic separation methods, i.e., GC (including GC × GC) and HPLC, have been discussed and their respective advantages and disadvantages highlighted. The use of sCO_2_ has great advantages compared to HD, such as the use of a low temperature, which prevents the formation of thermal artefacts, and the direct recovery of terpenes without using conventional organic solvents [25].

As expected, the major terpene compounds found in our sCO_2_ extracts were monoterpenes. From the results presented in Table 2, the main component was β-myrcene (15.55–33.45%), an acyclic monoterpene previously found most abundantly in numerous cannabis cultivars [24]. Thus, the studied sample of *C. sativa* can be assigned to the β-myrcene chemotype. Other known hemp chemotypes include α-pinene, limonene, terpinolene, linalool, β-caryophyllene, selina-3,7(11)-diene, γ-selinene, 10-epi-γ-eudesmol, β-eudesmol, α-eudesmol, bulnesol, and α-bisabolol [24]. Among the other monoterpenes, α-pinene (5.13–12.88%), β-pinene (2.94–7.78%), and limonene (2.99–6.65%) were the most abundant in the sCO_2_ extracts. A variety of other monoterpenes was found as a minor constituent. Numerous biological properties are attributed to the main monoterpenes in the study extracts. For example, β-myrcene had antipsychotic, sedative, muscle relaxant, analgesic, antioxidant, anti-inflammatory, and anticancerogenic properties [26,27,28]. In addition to potent antimicrobial and antiseptic effects, α-pinene also showed anti-inflammatory, bronchodilator, and gastroprotective effects [29]. Limonene showed antimicrobial and gastroprotective activities, but anxiolytic, antidepressant, antispasmodic, antiproliferative, and immunostimulatory activities were also highlighted [30,31]. 

Among the sesquiterpenes in the studied sCO_2_ extracts, β-caryophyllene was the most abundant (2.83–10.16%). It has been found to possess various biological activities, such as antimicrobial, antioxidant, anti-inflammatory, gastroprotective, analgesic, anticancerogenic, antiproliferative, antidepressant, anxiolytic, and neuroprotective activities [32,33]. As the only known terpene with this ability, β-caryophyllene interacts with the endogenous cannabinoid system and selectively binds to the CB2 receptor [34]. It also showed high selectivity against herpes simplex virus type 1 in vitro [35]. Table 2 shows that hydrocarbons (γ-selinene, selina-3,7(11)-diene) and alcohols (guaiol, γ-eudesmol, bulnesol), represented in concentrations ranging from 1.26 to 6.59%, also contribute to the sesquiterpene fraction of the studied extracts. Guaiol may act as an anti-inflammatory, antimicrobial, analgesic, and antitumor agent [36,37], while bulnesol has antitussive and expectorant properties [38]. 

Our research confirmed the previous findings that β-caryophyllene and β-myrcene are often the most abundant terpenes in *C. sativa* [24]. Due to their high content and various biological properties, they most likely have a significant impact on the medicinal outcomes of cannabis use. The cannabis extracts studied in the present work were also rich in α-pinene, β-pinene, guaiol, γ-eudesmol, and bulnesol, which may also contribute to their biological activities. Therefore, a possible synergistic and/or entourage effect of cannabinoids and terpenes should be considered [39], and further studies are needed to elucidate the possible interactions of cannabinoids and terpenes in humans [24]. 

The most abundant terpenes were used for further statistical analysis, and the summarized results of ANOVA of the models for each response studied are proposed (Table S2). Based on the obtained results, the regression models for all targeted terpenes were significant (*p* < 0.05), and the obtained R^2^ values ranged from 0.7532 to 0.9342 with non-significant fitting deficiencies, indicating an adequate representation between the input parameters and the observed variables. Moreover, an identical statistical effect can be seen for the monoterpenes (α-pinene, β-pinene, β-myrcene, and limonene) and guaiol with respect to the linear term of pressure as well as the quadratic term of temperature, which had a significant effect. For the sesquiterpenes, the quadratic term of pressure and temperature had a significant influence on β-caryophyllene abundance, whereas only the linear term of pressure had a significant influence on γ-eudesmol and bulnesol. For all components, the interaction between pressure and temperature had no effect. 

**Figure 2:**
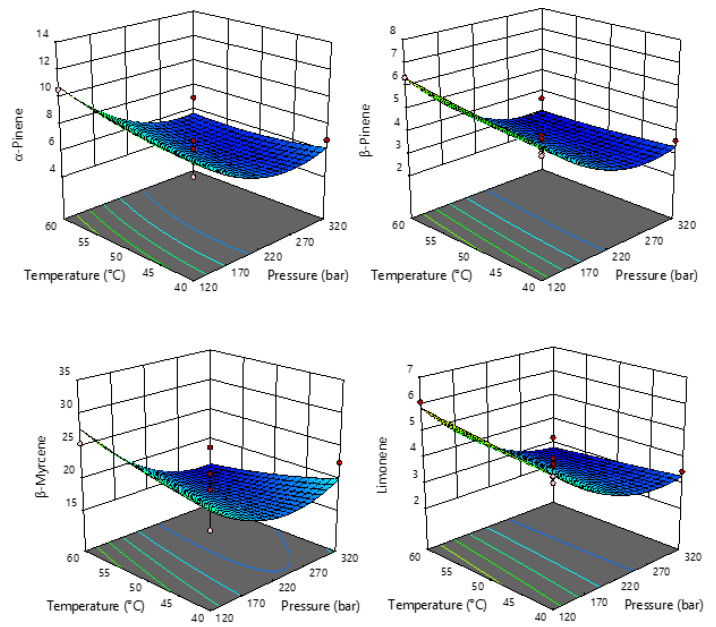
Response surface plots showing the effects of temperature and pressure on the selected monoterpenes.

**Figure 3:**
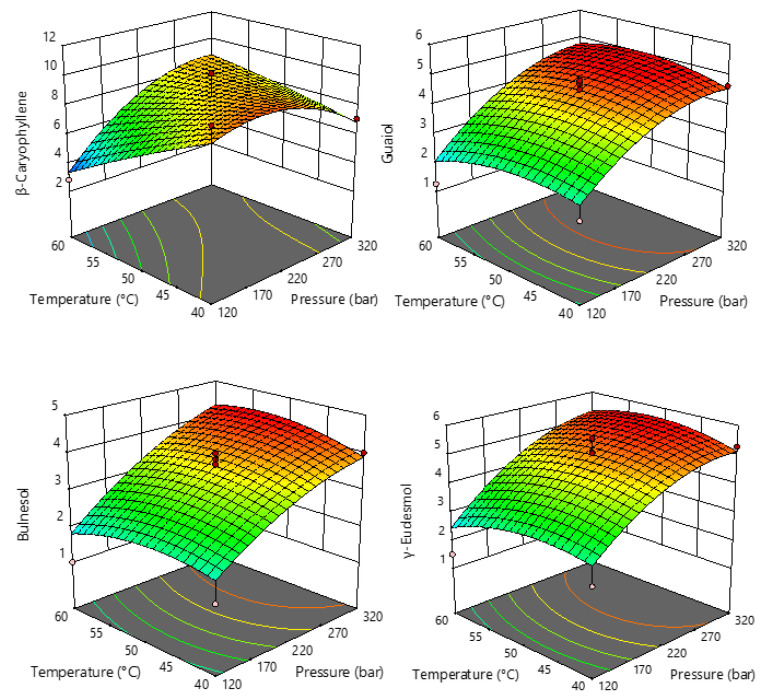
Response surface plots showing the effects of temperature and pressure on the selected sesquiterpenes.

It is interesting to see in Figure 2 and Figure 3 that the response surface plots for all monoterpenes have a similar shape, while all sesquiterpenes have a very similar shape of response plots. Monoterpenes and sesquiterpenes showed completely opposite effects of pressure on their abundance. In the case of monoterpenes (Figure 2), it is obvious that their content decreases with increasing pressure, so lower pressures are recommended for the extraction of monoterpenes.

From Figure 3, it can be seen that pressure has a strong influence and that the contents of guaiol, γ-eudesmol, and bulnesol increase at higher pressure, while the content of β-caryophyllene increases at pressures up to 300 bar and decreases slightly at higher pressures. Therefore, it can be concluded that the application of higher pressures favors the extraction of larger proportions of sesquiterpenes.

Compared to other studies using sCO_2_ extracts [20,24,25], there is a great similarity between the content and composition of the main terpene compounds. However, our design of sCO_2_ experiments was appropriate, since a great similarity of the obtained sCO_2_ extracts and the inflorescence extracts (Table 2) can be observed in terms of abundance of the main compounds and determination of chemotypes. This is a significant improvement over the previous study by Sexton et al. [40], which showed that the products of sCO_2_ extraction can have a significantly different chemotype fingerprint than the cannabis inflorescence.

### 2.3. Cannabinoids in sCO_2_ Extracts of Industrial Hemp

It is well known that *C. sativa* is the main source of cannabinoids; thus, the most important have been identified in sCO_2_ extracts, including CBD, CBDA, THC, CBC, CBG, and CBN. However, in the present study, some less represented cannabinoids were also analyzed by the HPLC-DAD, such as cannabigerolic acid (CBGA), tetrahydrocannabivarinic acid (THCVA), cannabichromene acid (CBCA), cannabidivaric acid (CBDVA), and tetrahydrocannabinolic acid A (THCA). Results are presented in Table 3, and the representative chromatograms of the HPLC analysis are given in the Supplementary Materials (Figure S1). In the context of cannabinoid analysis, it is important to understand the biosynthesis of cannabinoids, with CBGA being the major precursor for THCA and CBDA. At high temperatures, both acids tend to break down into their respective decarboxylated analogues, THC and CBD. When THC is oxidized in air and by light, CBN is formed, the result of the chemical decomposition of THC. The more THC that decomposes, the more CBN that is formed. CBN is present in old, dried hemp flowers. When consumed in large quantities, it can lead to paranoia. Therefore, proper storage of the plant is extremely important (hermetically sealed containers); this will extend its shelf life and slow down the natural process of CBN formation. At higher temperatures, the acidic forms are further converted into their neutral degradation products. Therefore, a heat-triggered chemical reaction leading to decarboxylation of these compounds is necessary if the corresponding CBD and THC are the target. All of the above forms possess some biological activity; thus, CBD and CBDA possess analgesic, antibacterial, antidiabetic, antiemetic, antiepileptic, anti-inflammatory, antiproliferative, and antipsychotic properties [41]. CBG is known for its analgesic, antibacterial, and antifungal effects, CBC exhibits anti-inflammatory, antibacterial, and antifungal activities, and CBN possesses sedative properties. The primary psychoactive substance THC has analgesic, anti-inflammatory, and antiemetic effects [4].

The main focus of this research was to obtain natural extracts rich in terpenes and CBD as a targeted cannabinoid. In our recently published review [42], sCO_2_ plant extracts showed their potential as bioactive ingredients for the development of effective and safe cosmetics. Nowadays, CBD is trending and is preferred in many cosmetic products due to its anti-inflammatory, analgesic, hydrating, moisturizing, and wrinkle–reducing properties [14]. It should be noted that more and more new cosmetic products are appearing on the market that contain only hemp extracts or CBD due to their strong nourishing effects on the skin. Previous research has shown that CBD with good carriers can penetrate the skin and prevent the signs of aging. This is because of the potential ability to protect against free radicals that cause oxidative damage in the skin, which in turn leads to visible damage, wrinkles, or the inevitable aging process of the skin. In addition to its anti-aging effect, the use of CBD products for skin problems such as acne, dermatitis, psoriasis, and other diseases is also being studied, as CBD acts on cellular processes that lead to acne, seborrhea, psoriasis, and dermatitis [1,14,43]. Therefore, these hemp extracts could be effective in treating certain skin problems; however, it is also known that sCO_2_ extracts can be excellent antioxidants for cosmetic products. Their addition offers numerous benefits for cosmetic products, mainly due to their antioxidant but also anti-inflammatory and antimicrobial effects. Moreover, CO_2_ in the supercritical state has been shown to be an extractant that ensures safe extracts for further cosmetic use [42,44].

For all these reasons, and especially because of the potential application of sCO_2_ extracts for topical use, CBD as a cannabinoid is targeted for further optimization processes. In addition, the presence of other cannabinoids in the extracts is also beneficial as they act synergistically. Therefore, CBDA is statistically evaluated as the most important cannabinoid. Table 3 lists eleven cannabinoids detected in the sCO_2_ extracts and inflorescences of industrial hemp. HPLC analysis showed that the cannabinoids studied were present in the crude plant material, with the exception of THCVA. It was expected that some of them would be present in very low concentrations (THC because industrial hemp contains only small amounts, CBN because it was a fresh sample and it was already mentioned that CBN is present in mature hemp flowers). As expected, CBDA dominated (14.02%), and CBD content in inflorescences was 0.59%.

It can also be seen from the results in Table 3 that the content of total cannabidiols (CBD+CBDA) extracted with sCO_2_ was very high. The CBDA content ranged from 6.32 to 30.63%, depending on the applied extraction parameters of pressure and temperature, while the CBD content ranged from 0.80 to 6.58%. 

**Figure 4:**
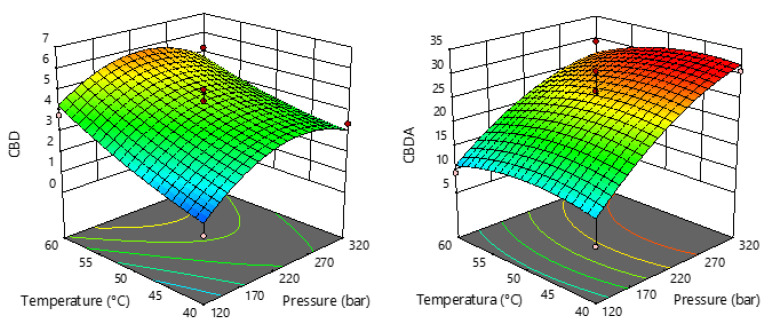
Response surface plots showing the effects of temperature and pressure on CBD and CBDA.

The statistical significance of the regression equations for the selected response (CBD and CBDA) was evaluated by ANOVA and is shown in Table S3. The regression models for both studied responses were highly significant according to *p*-values (0.0009 for CBD and 0.0072 for CBDA), with satisfactory coefficients of determination (R^2^). The non-significant lack of fit (*p* > 0.05) for each response indicates that the second order polynomial model is appropriate and can be used for the precision of the experimental values. It is also evident that the linear term of pressure and temperature, as well as the quadratic term of temperature, have a significant effect on the CBD content in the sCO_2_ extracts. In terms of CBDA content, only pressure had a statistically significant influence (*p* = 0.0006), which is also evident in Figure 4, where the trend shows that the higher the extraction pressure, the more CBDA that can be extracted.

Figure 4 also shows that higher temperature causes an increase in CBD content, while pressure has dual effects. An increase in pressure up to 250 bar resulted in an increase in CBD content, while pressures above 250 bar caused a slight decrease in content.

Kitrytė et al. [19] described that in sCO_2_ extracts, CBD (predominantly) and CBDA constituted about 28% of the extract, and 93% of the available CBD and CBDA were extracted. An increase in pressure resulted in a decrease in the content of cannabinoids in the extract. The highest content of cannabinoids was obtained in the extract at the lowest pressure (from 100 bar to 500 bar) and temperature (from 35 to 75 °C)—64.18 mg/g. Vági et al. [45] studied industrial hemp residues and concluded that an extraction pressure of 350 bar and a temperature of 45 °C were suitable parameters for the extraction of cannabinoids with high yields. Further increasing the pressure did not increase the extraction yield and amounts of cannabinoids in the extracts. A study by Rovetto and Aieta [10] showed that sCO_2_ extraction can be an excellent procedure for the extraction of cannabinoids from *C. sativa*, and the best results were obtained at 55 °C and 340 bar and without the addition of co-solvents. Omar et al. [46] studied the supercritical extraction of cannabinoids from 13 different samples of medicinal cannabis and analyzed the influence of the process parameters (from 100 bar to 250 bar, from 35 to 55 °C, with ethanol as co-solvent from 0 °C to 40%) on the extraction efficiency and quality. The CBD content in the extracts obtained in this way ranged from 1 mg/g to 13 mg/g, indicating that the plant material used is a good source of CBD. Perrotin-Brunel et al. [47] showed that the solubility of CBD in sCO_2_ increases with pressure. Interestingly, the highest solubility of CBD is obtained at a medium temperature (53 °C). This particular behavior is theoretically possible and has already been observed for the solubility of CBN in sCO_2_. The reason for this unusual behavior could be the transition from a solid–supercritical fluid equilibrium to a liquid–supercritical fluid equilibrium. The melting point of pure CBD (67 °C) and pure CBN (77 °C) is close to the experimental temperature of 61 °C. The melting depression effect of CO_2_ may have caused melting at 61 °C, resulting in lower solubility. 

### 2.4. Antiradical and Antibacterial Activity of Hemp sCO_2_ Extracts

**Figure 5:**
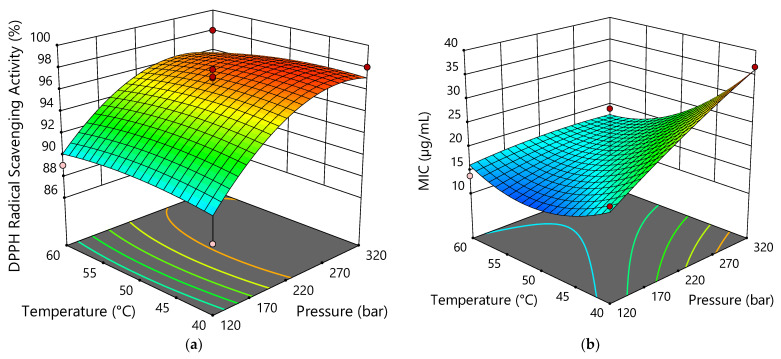
Response surface plots showing the effects of temperature and pressure on antiradical activity (**a**) and antibacterial activity (against *B. subtilis*) (**b**).

The antiradical activities of the sCO_2_ extracts at 250 µg mL^−1^ were evaluated by the DPPH scavenging assay, as shown in Table 4. Extract run 1 (320 bar; 40 °C), with the highest CBDA content of 30.68%, had the highest antiradical activity (98.06 ± 0.92%). The activity was comparable to extracts 7, 11, and 13 (220 bar; 50 °C). The lowest antiradical activity (87.56 ± 0.69%) was found for extract run 4 (120 bar; 40 °C), with a CBDA content of 6.32%. From these data, as well as data from ANOVA (Table S4), it appears that pressure had the greatest effect on antiradical activity, which is also evident from Figure 5. With the increase of pressure, better antioxidant activity is obtained.

Dawidowicz et al. [48] found that CBG, CBD, Δ9-THC, CBN, CBGA, CBDA, and Δ9-THCA have antioxidant activity due to their ability to scavenge free radicals, protect oxidation processes, and reduce metal ions. Extract run 1 also contained a high level of Δ9-tetrahydrocannabinolic acid (THCA-A) of 1.17%. McPartland et al. [49] suggested that THCA-A may be more stable in herbal cannabis, along with terpenes that serve as protective antioxidants and likely inhibit the oxidative decarboxylation of THCA to THC.

The sCO_2_ extracts were tested in vitro for antibacterial activity against *E. coli*, *P. aeruginosa*, *B. subtilis*, and *S. aureus.* The MIC values are shown in Table 5. 

All tested extracts exhibited good antibacterial activities against *E. coli*, *P. aeruginosa*, *B. subtilis*, and *S. aureus.* MIC values were between 10.42 µg mL^−1^ and 66.03 µg mL^−1^. The extracts were more active against Gram-positive bacteria than Gram-negative bacteria. The best antibacterial effect was observed against *B. subtilis* (these data were used for further analysis and optimization), while *P. aeruginosa* was the least sensitive to the action of the extracts. The main reason for the differences in bacterial susceptibility could be related to the presence of the outer membrane and lipopolysaccharide; Zhang et al. [50] found that membrane-disrupting drugs or LPS-deficient bacteria increased the susceptibility of Gram-negative bacteria to CBD. As previously reported, cannabinoids are thought to be responsible for the antimicrobial activity of hemp [51,52]. Among these compounds, CBD seems to be the most effective from a pharmaceutical point of view, although CBG and CBC, which are present in female hemp flowers, have remarkable antibacterial activity [53]. Appendino et al. [54] investigated the efficacy of five major cannabinoids (CBD, CBC, CBG, THC, and CBN) against six methicillin-resistant *S. aureus* (MRSA) strains of current clinical relevance and all showed MIC values in the range of 0.5–2 μg mL^−1^. The lowest MIC value against *S. aureus* was exhibited by extract run 6, which corresponds to a CBD content of 0.7 μg mL^−1^. In addition to cannabinoids, hemp volatile terpenes have been shown to be particularly important, such as α-pinene, β-myrcene and α-terpinolene, the most abundant compounds among the monoterpenes in our extracts, as well as β-caryophyllene and α-humulene as the main sesquiterpenes [51,52,55,56,57]. Extract run 6, with the highest CBD content of 67.15 µg mg^−1^, α-pinene content of 12.88 µg mg^−1^, β-pinene content of 7.78 µg mg^−1^, β-myrcene content of 33.45 µg mg^−1^, and limonene content of 6.65 µg mg^−1^ showed the best antibacterial activity, with a MIC value of 10.42 µg mL^−1^ against *B. subtilis*, *S. aureus,* and *E. coli* and twice the MIC value against *P. aeruginosa*. In run 6, the lowest pressure of 78.6 bar was used, confirming that lower pressures are desirable to obtain extracts with better antibacterial activity, which is also evident from Figure 5. 

The linear terms of pressure and temperature also showed a statistically significant effect on the antibacterial activity of the sCO_2_ extracts (data in Table S4). According to the results of the present study, CBD content and abundant terpenes appeared to influence the antibacterial activity of the extracts. Interestingly, in the study by Blašković et al. [58], CBD showed antibacterial activity against a broad spectrum of Gram-positive bacteria but was generally inactive against 20 Gram-negative bacterial strains, including *E. coli* and *P. aeruginosa.* Issepi et al. [59] found that pure CBD had no remarkable activity against *Staphylococcus* strains. However, some previous studies support our findings on the synergistic antibacterial activity of volatile terpenes and CBD. Nissen et al. [52] observed good antibacterial activity of CBD and monoterpenes (α-pinene, β-pinene, and β-myrcene), especially against *Listeria* and *Enterococcus* strains.

### 2.5. Optimization of Extraction Conditions

By optimizing the process parameters of pressure and temperature, it is possible to influence the properties of sCO_2_ and extract specific plant constituents, including those that are chemically sensitive, making this technique very efficient and selective [5,6]. The applied numerical optimization of sCO_2_ extraction of selected bioactive compounds with certain biological activities from the inflorescences of industrial hemp would allow the process to be carried out in the most efficient way, resulting in maximum output under minimum input conditions.

Our first optimization aimed to obtain extracts with higher extraction yield, rich in CBD, and with good antiradical and antibacterial activities. The maximum value was set for the extraction yield. The best antibacterial activity in the obtained sCO_2_ extracts was found to be against *B. subtilis*; the lower value was chosen as the target value in the numerical optimization, and the highest value was chosen for antiradical activity. As for the bioactive components themselves, CBD was included in the optimization to obtain its maximum content in the extracts (for possible topical application). The optimal conditions for all these selected conditions are shown in Table 6.

The second optimization was performed with the two target components, CBD and CBDA, since both have the desired bioactivity, and CBDA is known to tend to be degraded to the corresponding decarboxylated analogue CBD. It can be seen that very similar values for pressure and temperature were obtained in both cases.

The third optimization was performed to obtain extracts rich in monoterpenes (with a focus on the most abundant: α-pinene, β-pinene, β-myrcene, and limonene), and the fourth focused on extracts rich in sesquiterpenes. Table 6 shows that a temperature of approximately 60 °C was optimal for all terpenes, while the low pressure of 131.2 bar was optimal for the extracts rich in monoterpenes and 319.7 bar for the extracts rich in sesquiterpenes. In the discussion section, we explained that opposite effects of pressure were found for the different terpene groups. For the monoterpenes, it was obvious that their content decreased with increasing pressure, while it increased for the sesquiterpenes.

The final optimal extraction parameters with the expected lower desirability function (0.503), with many parameters included in the calculation, were determined for the case where a maximum of terpenes and cannabinoids are preferred in sCO_2_ extracts with high extraction yield and high antiradical and antibacterial activity. Omar et al. [46] also performed optimization experiments for marijuana samples, and two opposite trends were observed for terpenes and cannabinoids, leading to the conclusion that different optimal extraction conditions (in terms of extraction parameters) are required depending on the type of target compound. Finally, a pressure of 100 bar and a temperature of 35 °C were chosen as optimal parameters for the extraction of both terpenes and cannabinoids. In our study of industrial hemp, the temperature of 60 °C proved to be optimal for all selected optimization conditions. Thus, each sample is specific (variety, time of harvest, part of the plant from which the extraction is performed, drying method, etc.), and experimental tests must be performed for each sample before conclusions can be drawn.

## 3. Materials and Methods

### 3.1. Plant Material

Dried inflorescences of industrial hemp (*Cannabis sativa* L.) variety Futura 75 obtained from a Croatian company (Hemp Agro d.o.o., Selci) were studied. Preparation of the material for extraction included crushing (grinding) and sieving on a standard series of sieves (Retsch, Grindomix GM200, Germany) [60].

### 3.2. Chemicals

The standards of cannabinoids purchased as solutions were used for HPLC analysis as follows: cannabidiol (CBD) (Dr. Ehrenstorfer GmbH, Augsburg, Germany), 100 µg/mL in methanol; cannabidiolic acid (CBDA) (Dr. Ehrenstorfer GmbH, Augsburg, Germany), 100.0 µg/mL in acetonitrile; cannabidivarinic acid (CBDVA), 100.0 µg/mL in acetonitrile; cannabidiolic acid (CBDA) (Cerilliant Corporation, Texas, TX, USA), 500 µg/mL in acetonitrile; cannabigerolic acid (CBGA) (Cerilliant Corporation, Texas, TX, USA), 500 µg/mL in acetonitrile; tetrahydrocannabivarinic acid (THCVA) (Cerilliant Corporation, Texas, TX, USA), 500 µg/mL in acetonitrile; tetrahydrocannabinolic acid (THCA-A) (Cerilliant Corporation, Texas, TX, USA), 500 µg/mL in acetonitrile; cannabinol (CBN) (Dr. Ehrenstorfer GmbH, Augsburg, Germany), 100 µg/mL in methanol; cannabichromene (CBC) (Dr. Ehrenstorfer GmbH, Augsburg, Germany), 100 µg/mL in methanol; cannabigerol (CBG) (Dr. Ehrenstorfer GmbH, Augsburg, Germany), 100 µg/mL in methanol; and (-)-delta9-THC solution (THC) (Cerilliant Corporation, Texas, TX, USA), 1.0 mg/mL in methanol. All solvents and chemicals used were of analytical grade.

### 3.3. Supercritical CO_2_ Extraction (sCO_2_)

Experiments were performed in an sCO_2_ system described in detail elsewhere [61]. A total of 100 g of ground dried inflorescences of industrial hemp was added to the extraction vessel. Extracts were collected in previously weighed glass tubes using a balance with an accuracy of ±0.0001 g. The extraction process took 30 min (determined as optimal time in preliminary studies). Separation conditions were 15 bar and 25 °C. The sCO_2_ extraction was performed under different pressure and temperature conditions, with a mass flow rate of 1.4 kg/h.

#### Statistical Experimental Design

The Central Composite Design (CCD) explained by Bas and Boyaci [62] was used to determine the optimal extraction conditions, pressure (*X*_1_), and temperature (*X*_2_) under which the highest content of desired constituents is obtained. The commercial software Design-Expert^®^ (ver. 9. Stat-Ease Inc., Minneapolis, MN, USA) was used to analyze the obtained results. In addition, the quality of the fitted model was evaluated using the analysis of variance (ANOVA), while the test of statistical differences was based on the total error with a confidence level of 95.0%.

### 3.4. HPLC Characterization of Cannabinoids

A total of 10 µg ± 2 µg of total sCO_2_ extract was dissolved in 1 mL of propan-2-ol. Then, 100 μL of this solution was taken and diluted to 2 mL with methanol. For analyses, samples were filtered through a syringe filter (13 mm) with a pore size of 0.45 µm into a vial. For comparison with the sCO_2_ extracts, analysis of the crude material (hemp inflorescences) was performed by extracting 1 g of plant material in 20 mL of solvent (acetonitrile-methanol, 55:45% *v*/*v*) for 1 h at 40 °C on a magnetic stirrer. The mixture was filtered, and 100 µL of the obtained extract was diluted to 2 mL with methanol and then filtered in the same manner as described.

HPLC analysis of cannabinoids was performed on an Agilent 1260 Infinity II (Agilent, Santa Clara, CA, USA) with chromatographic separation on an InfinityLab Poroshell 120-C18 (4.6 mm × 150 mm, 4 µm). The separation of the analyzed compounds was performed by gradient elution at a flow rate of 1 mL/min for 67 min, using 0.1% formic acid in Millipore water (Millipore Simplicity 185, Darmstadt, Germany) as phase A and 0.05% formic acid in methanol as phase B. The gradient was set as follows: 0.00–7.05 min 40% A; 7.05–49.37 min 23% A; 49.37–67.00 min 5% A, followed by a post run time of 10 min during which conditions were reset to baseline. The injection volume was 35 µL, UV detection wavelengths were 210 nm and 230 nm, and analyses were performed at 50 °C. Cannabinoid identification was based on retention time and comparison of the absorbance spectrum in the extracts with the spectrum of standard components, while quantification was based on external calibration. The retention time for CBDVA was 23.236 min, for CBD 31.793 min, for CBG 33.123 min, for CBDA 35.253 min, for CBGA 41.228 min, for CBN 43.252 min, for THC 47.047 min, for THCVA 48.210 min, for CBC 55.261 min, for THCA 60.492 min, and for CBCA 62.358 min.

The standard stock solutions for cannabinoids were prepared in a solvent (methanol), and calibration was performed at eight concentrations, depending on the ingredients. The standard stock solution for CBDVA was prepared in the range of 1.25–125.0 µg/mL, for CBD 5.00–100 µg/mL, for CBG 1.875–75.00 µg/mL, for CBDA 1.00–50.00 µg/mL, for CBGA 1.25–125.00 µg/mL, for CBN 1.00–50.00 µg/mL, for THC 3.125–50.00 µg/mL, for THCVA 1.25–125.00 µg/mL, for CBC 1.875–75.00 µg/mL, for THCA 1.25–125.00 µg/mL, and for 1.25–125.00 µg/mL. The linearity of the calibration curve was confirmed by R^2^ = 0.99996 for CBDVA, R^2^ = 0.99868 for CBD, R^2^ = 0.99913 for CBG, R^2^ = 0.99921 for CBDA, R^2^ = 0.99995 for CBGA, R^2^ = 0.99910 for CBN, R^2^ = 0.99995 for THC, R^2^ = 0.99996 for THCVA, R^2^ = 0.99810 for CBC, R^2^ = 0.99994 for THCA, and R^2^ = 0.99996 for CBCA.

### 3.5. Gas Chromatography-Mass Spectrometry (GC-MS) Analysis of Volatile Compounds

A total of 10 µg ± 2 µg of total sCO_2_ extract was dissolved in 1 mL hexane and filtered through a premium syringe filter (13 mm) with polytetrafluoroethylene membrane and 0.45 µm pore size into a 2 mm brown GC vial N 9. The extract of the crude material (hemp inflorescence) was prepared for comparison with the sCO_2_ extracts. Then, 1 g of the plant material was macerated in 5 mL hexane for 2 h and filtered by the same procedure as described above. The prepared samples were analyzed using an Agilent 7890B gas chromatograph coupled to a mass spectrometer Agilent, series 5977A. The volatile compounds of the extracts were separated on a HP-5MS capillary column (30 m × 0.25 mm, 0.25 μm) (19091 S - 433 UI-INT, Agilent Technologies, Santa Clara, CA, USA). The injector temperature was set at 250 °C, and a 3 μL sample was injected in a 1:50 split mode. Helium with a purity of 99.99% was used as the carrier gas at a constant flow rate of 1 mL/min. The following temperature program was set: 70 °C (2 min), which was increased by 3 °C/min up to 200 °C and kept constant for 18 min. The separated components were analyzed by mass spectrometry (electron energy of 70 eV) with a scan range *m*/*z* 30–450. The temperature of the ion source was 230 °C, while the temperature of the quadrupole was 150 °C. The analysis of each sample was performed in three replicates.

### 3.6. Determination of Antiradical Activity of sCO_2_ Extracts

The antiradical activity of the extracts was determined using the DPPH radical scavenging assay described previously [63]. Then, 750 μL of diluted extracts (final concentration 250 µg mL^−1^) were mixed with an equal amount of a 0.2 mM DPPH radical solution so that the final DPPH radical concentration was 0.1 mM. The mixture was stirred well and incubated at room temperature for 30 min. The absorbance decrease was measured at 517 nm. Ascorbic acid (AA) was used as a reference compound in the concentration range of 2–200 μg mL^−1^. All experiments were performed in triplicate.

### 3.7. Determination of Antibacterial Activity of sCO_2_ Extracts

The determination of minimal inhibitory concentrations (MIC) of obtained extracts was performed by the modified broth microdilution method in Mueller Hinton Broth (Fluka, BioChemica, Germany) according to the Clinical Laboratory and Standard Institute (CLSI) M7-A7 document [64]. Bacteria were collected from different clinical samples obtained from the Microbiological Service of the Public Health Institute of Osijek-Baranja County, Croatia. *Bacillus subtilis*, *Staphylococcus aureus*, *Escherichia coli,* and *Pseudomonas aeruginosa* previously cultured on Mueller Hinton agar were suspended in saline at a density standardized to 0.5 McFarland standard turbidity and added to 100 μL of two-fold serially diluted obtained extracts. Each plate contained a growth control (bacterial inoculum without extracts), background control (broth and ethanol), and the antibacterial standard ciprofloxacin (Hospira, Hurley, Maidenhead, Berkshire, England, UK). After incubation at 37 °C for 24 h, another incubation at 37 °C for three hours was performed using triphenyltetrazolium chloride as a reducing agent indicator for microbial growth. The MIC was defined as the lowest extract concentration at which no color change due to microbial growth occurred, derived from triplicate analyses and normalized against the negative control.

## 4. Conclusions

Natural pharmaceuticals containing terpenes and especially cannabinoids are in great demand and are a hot topic today. In the present work, “green” extraction with sCO_2_ was successfully used to extract terpenes and cannabinoids from the inflorescences of industrial hemp. The GC-MS analysis of the sCO_2_ extracts revealed the chemotype of β-myrcene, with abundant α-pinene and β-pinene and a variety of other monoterpenes as minor constituents, while the most abundant sesquiterpene was β-caryophyllene. The main cannabinoids identified in the sCO_2_ extracts were CBDA and CBD, followed by minor amounts of CBCA, CBC, THCA-A, THC, CBGA, CBG, and CBDVA, while CBN and THCVA were not detected. The extract with the highest CBDA content, obtained at 320 bar and 40 °C, exhibited the best antiradical properties. All tested extracts showed good antibacterial activities against *E. coli*, *P. aeruginosa, B. subtilis*, and *S. aureus*. The sCO_2_ extract with the highest CBD content, containing abundant α-pinene, β-pinene, β-myrcene, and limonene, showed the best antibacterial activity. The optimal conditions for sCO_2_ extraction of cannabinoids and terpenes from industrial hemp were established. The temperature of 60 °C was found to be optimal for all responses studies, while the pressure showed a different effect depending on the target substance. Low pressure was optimal for monoterpenes, while higher pressure was optimal for the extracts rich in sesquiterpenes. In addition, high pressure was also beneficial for CBD.

In the proposed research work, the sCO_2_ extraction process was optimized to obtain the desired extracts of *C. sativa,* rich in the target constituents with antiradical and antibacterial properties. This environmentally friendly technological process could be implemented in a production system for the extraction of cannabinoids and terpenes with the aim of their topical application for medicinal and cosmetic purposes in a safe and effective way.

## Figures and Tables

**Table 1:** CCD matrix (levels of independent variables) for further sCO_2_ optimization.

Run	Pressure (Bar) *X*_1_	Temperature (°C) *X*_2_	Extraction Yield (%) Y
1	320	40	6.16
2	220	35.9	5.67
3	220	50	5.53
4	120	40	3.20
5	220	64.1	6.39
6	78.6	50	0.75
7	220	50	5.85
8	320	60	8.83
9	120	60	2.31
10	361.4	50	8.79
11	220	50	5.80
12	220	50	5.62
13	220	50	5.39

**Table 2:** Content and composition of volatile compounds in hemp sCO_2_ extracts (%).

No.	Compound	RI	1	2	3	4	5	6	7	8	9	10	11	12	13	Inflorescences
1	Hexan-1-ol	875	0.06	0.06	0.08	0.09	0.10	0.06	0.09	0.08	0.11	0.09	0.09	0.08	0.09	0.06
2	Heptanal	903	0.17	0.10	0.10	0.04	0.10	0.04	0.14	0.11	0.04	0.16	0.09	0.13	0.12	0.14
3	α-Thujene	934	-	-	-	-	-	0.02	-	-	0.01	-	-	-	-	0.01
4	α-Pinene	941	6.82	6.46	6.03	7.89	6.41	12.88	6.77	6.94	10.58	5.14	6.12	5.13	5.55	6.81
5	Camphene	956	0.15	0.15	0.16	0.11	0.16	0.40	0.17	0.17	0.32	0.12	0.16	0.13	0.15	0.16
6	Sabinene	980	-	-	-	-	-	-	-	-	-	-	-	-	-	0.03
7	β-Pinene	983	3.59	3.55	3.66	5.11	3.40	7.78	3.85	3.63	6.38	2.94	3.70	3.02	3.23	3.41
8	β-Myrcene	993	22.47	18.40	18.24	19.98	18.31	33.45	20.70	18.47	25.44	15.55	17.29	15.66	15.65	16.75
9	α-Phellandrene	1007	0.02	0.03	0.04	0.06	0.03	0.09	0.04	0.05	0.04	0.06	0.06	0.03	0.03	0.04
10	Δ-3-carene	1014	0.03	0.02	0.03	0.05	0.03	0.06	0.03	0.04	0.04	0.04	0.05	0.02	0.02	0.03
11	α-Terpinene	1021	0.02	-	0.03	0.04	0.03	0.05	0.03	0.04	0.03	0.05	0.05	0.02	0.02	0.03
12	Limonene	1033	3.44	3.42	3.67	5.04	3.28	6.65	3.97	3.19	6.08	2.89	3.75	2.99	3.23	2.71
13	1,8-Cineole	1036	0.02	0.02	0.03	0.01	0.02	0.01	0.03	0.02	0.01	0.04	0.02	0.02	0.02	0.01
14	(*Z*)-β-Ocimene	1041	0.04	0.04	0.06	0.09	0.06	0.13	0.07	0.05	0.10	0.05	0.06	0.04	0.05	0.03
15	(*E*)-β-Ocimene	1052	1.24	1.28	1.38	2.08	1.34	3.10	1.61	1.11	2.36	1.11	1.51	1.20	1.21	1.04
16	γ-Terpinene	1063	0.03	0.03	0.03	0.04	0.03	0.04	0.04	0.05	0.04	0.05	0.05	0.03	0.03	0.04
17	*cis*-Sabinenehydrate	1071	0.05	0.05	0.07	0.06	0.07	0.03	0.07	0.06	0.05	0.06	0.07	0.07	0.06	0.06
18	Octan-1-ol	1072	0.03	0.04	0.06	0.03	0.07	0.03	0.07	0.07	0.03	0.10	0.06	0.07	0.07	0.07
19	*trans*-Linalool oxide	1076	0.03	0.03	0.04	0.04	0.04	0.02	0.05	0.04	0.03	0.05	0.05	0.04	0.05	0.03
20	α-Terpinolene	1091	0.78	0.56	0.92	1.41	0.61	1.12	0.79	0.60	0.97	0.75	1.07	0.58	0.55	0.62
21	Linalool	1100	0.04	0.07	0.07	0.08	0.07	0.05	0.07	0.07	0.08	0.07	0.07	0.08	0.09	0.07
22	Nonanal	1105	0.13	0.05	0.08	0.05	0.09	0.03	0.08	0.12	0.03	0.14	0.07	0.13	0.08	0.16
23	Hexyl propanoate	1107	0.04	0.04	0.06	0.10	0.04	0.06	0.05	0.05	0.06	0.04	0.06	0.03	0.05	0.00
24	Fenchol	1115	0.52	0.61	0.64	0.65	0.66	0.33	0.68	0.57	0.55	0.62	0.68	0.66	0.65	0.54
25	*trans*-Pinocarveol	1142	-	0.02	-	-	-	-	-	-	-	0.02	-	-	-	0.00
26	Borneol	1169	0.17	0.18	0.21	0.17	0.21	0.10	0.24	0.20	0.12	0.22	0.23	0.22	0.23	0.18
27	Terpinen-4-ol	1179	0.03	0.03	0.04	0.03	0.04	0.02	0.04	0.04	0.02	0.05	0.05	0.04	0.05	0.04
28	α-Terpineol	1191	0.03	0.05	0.07	0.05	0.06	0.03	0.07	0.07	0.03	0.07	0.08	0.08	0.07	0.06
29	Hexyl butanoate	1193	0.60	0.66	0.73	0.91	0.62	0.52	0.79	0.51	0.96	0.54	0.77	0.67	0.63	0.48
30	β-Citronellol	1230	0.04	0.04	0.06	0.06	0.07	0.02	0.07	0.06	0.04	0.07	0.08	0.07	0.07	0.07
31	1H-Indole	1294	-	-	-	-	-	-	-	-	-	-	-	-	-	0.03
32	α-Ylangene	1373	0.07	0.09	0.09	0.05	0.10	0.04	0.09	0.09	0.11	0.10	0.09	0.09	0.07	0.08
33	α-Copaene	1376	0.03	0.03	0.04	0.03	0.04	0.02	0.04	0.05	0.04	0.05	0.05	0.04	0.05	0.06
34	Hexyl hexanoate	1388	0.10	0.11	0.11	0.14	0.12	0.04	0.14	0.08	0.12	0.10	0.15	0.13	0.11	0.08
35	α-Gurjunene	1408	0.03	0.03	0.03	0.03	0.03	0.02	0.04	0.02	0.04	0.04	0.04	0.03	0.03	0.03
36	*cis*-α-Bergamotene	1415	0.07	0.09	0.09	0.11	0.09	0.05	0.09	0.07	0.12	0.09	0.09	0.08	0.11	0.08
37	β-Caryophyllene	1419	7.09	8.53	8.45	9.88	8.34	3.94	10.16	7.49	2.83	7.56	8.31	7.99	8.15	7.18
38	γ-Elemene	1434	0.33	0.35	0.43	0.54	0.34	0.11	0.45	0.31	0.29	0.33	0.42	0.40	0.36	0.25
39	*trans-*α-Bergamotene	1437	0.53	0.61	0.60	0.75	0.58	0.29	0.62	0.50	0.78	0.52	0.59	0.58	0.66	0.48
40	α-Guaiene	1439	0.02	0.05	0.06	0.06	0.06	0.02	0.05	0.05	0.05	0.05	0.06	0.06	0.06	0.04
41	3,7-Guaiadiene	1444	0.05	0.11	0.10	0.13	0.10	0.04	0.10	0.08	0.12	0.10	0.11	0.10	0.10	0.08
42	α-Humulene	1454	2.23	2.78	2.79	3.31	2.75	1.09	2.83	2.42	3.15	2.50	2.76	2.67	2.68	2.31
43	*trans*-β-Farnesene	1460	1.26	1.53	1.61	1.92	1.54	0.64	1.65	1.25	1.91	1.39	1.62	1.50	1.61	1.24
44	α-Amorphene	1466	0.02	0.04	0.04	0.05	0.06	0.04	0.05	0.05	0.05	0.05	0.05	0.04	0.06	0.06
45	γ-Muurolene	1476	0.24	0.32	0.31	0.38	0.33	0.12	0.34	0.30	0.32	0.33	0.34	0.34	0.33	0.28
46	β-Eudesmene	1483	0.11	0.09	0.11	0.22	0.43	0.03	0.12	0.35	0.39	0.39	0.13	0.11	0.14	0.34
47	β-Selinene	1485	0.96	1.15	1.16	1.32	1.19	0.38	1.19	1.02	1.07	1.07	1.19	1.14	1.12	0.95
48	Valencene	1492	0.46	0.55	0.57	0.65	0.58	0.17	0.58	0.48	0.53	0.53	0.59	0.52	0.53	0.00
49	α-Selinene	1493	0.91	1.10	1.11	1.24	1.13	0.35	1.14	0.98	1.00	1.02	1.13	1.10	1.05	1.38
50	(*E*,*E*)-α-Farnesene	1509	2.10	2.55	2.67	3.28	2.49	0.79	2.71	2.09	3.02	2.34	2.64	2.47	2.44	1.98
51	γ-Cadinene	1515	0.18	0.22	0.18	0.16	0.19	0.09	0.20	0.19	0.14	0.21	0.19	0.20	0.22	0.18
52	Bicyclogermacrene	1517	0.67	0.83	0.83	0.92	0.84	0.67	0.85	0.72	0.70	0.75	0.85	0.82	0.79	0.68
53	β-Cadinene	1518	0.51	0.63	0.60	0.68	0.66	0.26	0.65	0.58	0.55	0.59	0.62	0.67	0.62	0.58
54	δ-Cadinene	1524	0.46	0.60	0.57	0.67	0.61	0.18	0.62	0.54	0.52	0.59	0.62	0.61	0.61	0.00
55	γ-Selinene	1535	3.49	4.28	4.11	4.60	4.46	1.34	4.43	4.01	3.51	4.05	4.26	4.59	4.21	4.12
56	Selina-3,7(11)-diene	1541	4.98	6.13	5.84	6.49	6.31	1.92	1.99	5.67	4.82	5.68	6.05	6.59	5.98	5.69
57	(*E*)-α-Bisabolene	1549	0.69	0.88	0.84	0.93	0.73	0.27	0.83	0.73	0.72	0.80	0.83	0.90	0.78	0.75
58	Germacrene B	1556	1.90	1.88	2.17	2.70	1.86	0.59	2.28	1.65	1.50	1.72	2.14	2.05	1.82	1.31
59	Nerolidol	1566	0.21	0.21	0.38	0.31	0.40	0.12	0.41	0.35	0.16	0.41	0.43	0.44	0.44	0.37
60	Caryophyllene oxide	1581	0.60	0.72	0.76	0.67	0.77	0.31	0.77	0.69	0.33	0.73	0.80	0.78	0.80	0.75
61	Guaiol	1601	4.62	3.96	3.94	2.03	4.28	2.32	4.51	4.54	1.26	4.85	4.51	4.80	4.68	4.87
62	Ledol	1599	0.13	0.12	0.11	0.13	0.12	0.08	0.12	0.12	0.04	0.14	0.12	0.12	0.11	0.13
63	γ-Eudesmol	1623	5.27	4.48	4.43	2.36	4.66	3.03	4.92	5.14	1.50	5.35	5.05	5.55	5.07	5.53
64	10-epi-γ Eudesmol	1630	0.76	0.63	0.70	0.31	0.29	0.19	0.30	0.32	0.20	0.36	0.31	0.33	0.33	0.38
65	Dihyro-*cis*-α-copaene-8-ol **	1634	0.26	0.23	0.11	0.12	0.27	0.17	0.28	0.31	0.08	0.34	0.29	0.11	0.31	0.34
66	Hinesol	1637	0.11	0.11	0.29	0.04	0.11	0.08	0.12	0.13	0.03	0.01	0.06	0.30	0.12	0.16
67	β-Eudesmol	1649	2.21	1.77	1.92	0.85	1.89	1.26	2.00	2.21	0.55	2.37	2.05	2.17	2.07	2.53
68	α-Eudesmol	1652	2.71	2.13	2.28	1.02	2.27	1.51	2.40	2.69	0.67	2.85	2.44	2.59	2.49	2.53
69	Bulnesol	1666	4.01	3.19	3.06	1.52	3.58	2.35	3.69	4.21	1.00	4.37	3.70	3.99	3.83	4.73
70	α-Bisabolol	1683	1.89	1.83	1.83	0.90	1.61	1.00	1.90	2.01	0.51	2.20	2.01	2.10	2.07	2.09
71	Juniper camphor	1692	0.44	0.39	0.43	0.18	0.43	0.27	0.46	0.56	0.14	0.60	0.46	0.48	0.46	0.65
72	Hexahydrofarnesyl acetone	1846	0.06	0.10	0.13	0.13	0.14	0.04	0.17	0.13	0.05	0.16	0.16	0.16	0.17	0.23
73	Heneicosane	2100	0.01	0.03	0.06	0.01	0.04	0.01	0.05	0.04	0.01	0.05	0.05	0.06	0.05	0.10
74	Phytol	2116	0.02	0.01	0.22	0.05	0.01	0.01	0.24	0.20	0.03	0.37	0.29	0.01	0.34	0.23

**: tentatively identified.

**Table 3:** Content of cannabinoids in different extraction runs of industrial hemp (according to CCD) expressed as percentage ± SD (%).

Run *	CBCA	CBG	CBN	THC	CBDVA	CBD	CBDA	CBGA	THCVA	CBC	THCA-A	Total CBD	Total THC
1	1.98 ± 0.03	0.55 ± 0.03	0.00	0.66 ± 0.03	0.20 ± 0.01	3.39 ± 0.07	30.68 ± 0.21	0.23 ± 0.01	0.00	1.21 ± 0.01	1.17 ± 0.03	30.21 ± 0.00	3.69 ± 0.05
2	0.03 ± 0.00	0.27 ± 0.01	0.00	0.65 ± 0.01	0.29 ± 0.00	3.45 ± 0.15	27.48 ± 0.09	0.51 ± 0.01	0.00	1.12 ± 0.00	0.05 ± 0.01	27.54 ± 0.00	0.69 ± 0.03
3	1.15 ± 0.01	0.43 ± 0.00	0.00	0.65 ± 0.01	0.00	4.42 ± 0.02	21.23 ± 0.03	0.11 ± 0.00	0.00	1.43 ± 0.01	1.47 ± 0.01	23.04 ± 0.01	1.93 ± 0.03
4	0.33 ± 0.00	0.29 ± 0.03	0.00	0.22 ± 0.00	0.00	0.80 ± 0.05	6.32 ± 0.07	0.00	0.00	0.61 ± 0.02	1.45 ± 0.02	6.34 ± 0.01	1.49 ± 0.02
5	0.05 ± 0.00	0.69 ± 0.01	0.00	0.90 ± 0.00	0.14 ± 0.00	6.58 ± 0.09	17.71 ± 0.22	0.17 ± 0.00	0.00	1.23 ± 0.02	0.06 ± 0.01	22.12 ± 0.00	0.95 ± 0.01
6	0.60 ± 0.02	0.44 ± 0.05	0.00	0.44 ± 0.02	0.11 ± 0.00	1.71 ± 0.02	11.08 ± 0.07	0.00	0.00	1.13 ± 0.01	0.60 ± 0.04	16.44 ± 0.02	0.97 ± 0.00
7	1.79 ± 0.03	0.68 ± 0.00	0.00	0.89 ± 0.00	0.17 ± 0.00	5.01 ± 0.11	30.63 ± 0.12	0.17 ± 0.00	0.00	1.42 ± 0.01	1.45 ± 0.06	31.87 ± 0.01	2.43 ± 0.05
8	0.00	0.71 ± 0.01	0.00	1.02 ± 0.01	0.29 ± 0.02	5.22 ± 0.04	29.30 ± 0.09	0.45	0.00	1.29 ± 0.02	1.04 ± 0.03	30.92 ± 0.01	1.93 ± 0.04
9	0.01 ± 0.00	0.56 ± 0.01	0.00	0.41 ± 0.01	0.00	3.78 ± 0.05	9.30 ± 0.26	0.00	0.00	0.75 ± 0.00	0.05 ± 0.02	11.93 ± 0.00	0.46 ± 0.04
10	0.00	0.37 ± 0.01	0.00	0.44 ± 0.01	0.00	2.01 ± 0.02	30.64 ± 0.02	0.00	0.00	0.67 ± 0.02	0.55 ± 0.02	15.73 ± 0.00	0.92 ± 0.03
11	1.42 ± 0.02	0.54 ± 0.02	0.00	0.71 ± 0.02	0.14 ± 0.00	3.97 ± 0.02	24.27 ± 0.05	0.14 ± 0.00	0.00	1.13 ± 0.01	1.38 ± 0.05	25.25 ± 0.01	1.92 ± 0.01
12	1.11 ± 0.00	0.52 ± 0.02	0.00	0.76 ± 0.02	0.00	4.41 ± 0.06	25.58 ± 0.24	0.29 ± 0.02	0.00	1.10 ± 0.00	1.27 ± 0.02	26.84 ± 0.00	1.87 ± 0.06
13	1.37 ± 0.03	0.53 ± 0.03	0.00	0.72 ± 0.03	0.00	4.47 ± 0.01	26.50 ± 0.12	0.20 ± 0.02	0.00	1.42 ± 0.01	1.31 ± 0.02	27.71 ± 0.00	1.87 ± 0.04
Inflorescences	1.59 ± 0.02	0.12 ± 0.00	0.19 ± 0.01	0.18 ± 0.03	0.11 ± 0.00	0.59 ± 0.03	14.02 ± 0.07	0.32 ± 0.01	0.00	0.28 ± 0.02	0.40 ± 0.01	30.21 ± 0.03	3.69 ± 0.05

* 1–13 => % of components in SFE extracts, inflorescences => % of components in plant material (conventional extraction). Total THC is the sum of percentage of THCA multiplied by 0.877 plus the percentage of THC presented in extract.

**Table 4:** Antioxidant activity of hemp sCO_2_ extracts expressed as % DPPH (2,2-diphenyl-1-picrylhydrazyl) radical scavenging activity at 250 µg mL^−1^.

Run	DPPH Radical Scavenging Activity (%)
1	98.06 ± 0.92
2	96.85 ± 1.37
3	95.58 ± 0.57
4	87.56 ± 0.69
5	94.17 ± 1.12
6	89.00 ± 1.87
7	97.85 ± 0.29
8	97.99 ± 0.92
9	89.07 ± 0.97
10	93.28 ± 0.87
11	97.20 ± 0.39
12	94.93 ± 0.54
13	97.09 ± 1.08
AA	78.77 ± 2.40

Data expressed as mean ± S.D. AA—ascorbic acid as reference compound.

**Table 5:** Minimum inhibitory concentrations (MIC) of hemp sCO_2_ extracts against *Escherichia coli*, *Pseudomonas aeruginosa, Bacillus subtilis*, and *Staphylococcus aureus* (µg mL^−1^).

Sample	MIC (µg mL^−1^)			
	*B. subtilis*	*S. aureus*	*E. coli*	*P. aeruginosa*
1	36.64	36.64	18.32	36.64
2	33.01	33.02	33.02	66.03
3	28.11	28.11	28.11	28.11
4	19.20	38.41	19.20	19.20
5	23.44	23.44	23.44	46.88
6	10.42	10.42	10.42	20.84
7	17.59	29.59	29.59	29.59
8	14.67	58.70	29.35	29.35
9	13.79	27.58	13.79	13.79
10	24.95	24.95	24.95	24.95
11	16.74	33.48	33.48	33.48
12	17.08	34.16	34.16	34.16
13	12.02	48.09	24.05	24.05
C	0.781	0.781	0.781	1.563

1–13: extract run; C: ciprofloxacin.

**Table 6:** Optimal sCO_2_ extraction conditions for extracts of certain characteristics.

Target	Pressure (Bar)	Temperature (°C)	Desirability
High extraction yield, high CBD, high antioxidative activity, minimum MIC	284.78	60	0.817
High extraction yield, high CBD and CBDA, high antioxidative activity, minimum MIC	294.1	58.9	0.815
High extraction yield, high monoterpenes, high antioxidative activity, minimum MIC	131.2	60	0.521
High extraction yield, high sesquiterpenes, high antioxidative activity, minimum MIC	319.7	58.2	0.892
High extraction yield, high terpenes and cannabinoids (most abundant), high antioxidative activity, minimum MIC	166.9	55.7	0.503

## Data Availability

Data is contained within the article and Supplementary Materials.

## Supplementary Materials

The following supporting information can be downloaded at: https://www.mdpi.com/article/10.3390/ph15091117/s1. Figure S1: Representative chromatogram of the HPLC analysis of the extracts number 4. Table S1: Analysis of variance (ANOVA) for the response surface quadratic models for extraction yield. Table S2: Analysis of variance (ANOVA) for the response surface quadratic models for selected terpenes. Table S3: Analysis of variance (ANOVA) for the response surface quadratic models for CBD and CBDA. Table S4: Analysis of variance (ANOVA) for the response surface quadratic models for antioxidant and antibacterial activity.
