# Supplementary material for: Terpenes and Cannabinoids in Supercritical CO2 Extracts of Industrial Hemp Inflorescences: Optimization of Extraction, Antiradical and Antibacterial Activity
Source: Pharmaceuticals (Basel). 2022 Sep 7;15(9):1117. doi: 10.3390/ph15091117 (PMC9502342; doi:10.3390/ph15091117)
Supplement: Supplementary file 1 [file pharmaceuticals-15-01117-s001.zip › pharmaceuticals-1870414-supplementary.pdf]

Supplementary Material for

Terpenes and Cannabinoids in Supercritical CO_2 Extracts of Industrial Hemp Inflorescences: Optimization of Extraction, Antiradical and Antibacterial Activity

Stela Jokić[1], Igor Jerković[2*], Valentina Pavić[3], Krunoslav Aladić[1], Maja Molnar[1], Martina Jakovljević Kovač[1] and Sanda Vladimir-Knežević[4]

[1] Faculty of Food Technology, Josip Juraj Strossmayer University of Osijek, Franje Kuhača 18, 31000 Osijek, Croatia
[4] Faculty of Chemistry and Technology, University of Split, Ruđera Boškovića 35, 21000 Split, Croatia
[3] Department of Biology, Josip Juraj Strossmayer University of Osijek, Cara Hadrijana 8/A, 31000 Osijek, Croatia
[4] Faculty of Pharmacy and Biochemistry, University of Zagreb, A. Kovačića 1, 10000 Zagreb, Croatia
* Correspondence: igor@ktf-split.hr; Tel.: +385-21-329-461

Content of listed material

Table S1. Analysis of variance (ANOVA) for the response surface quadratic models for extraction yield.

Source	Sum of squares	df	Mean square	F-value	p-value *
			Extraction yield		
Model	60.50	5	12.10	104.76	< 0.0001
X_1	54.33	1	54.33	470.38	< 0.0001
X_2	0.9855	1	0.9855	8.53	0.0223
X_1^2	3.15	1	3.15	27.31	0.0012
X_2^2	1.77	1	1.77	15.32	0.0058
X_1X_2	0.1119	1	0.1119	0.9685	0.3578
Residual	0.8085	7	0.1155		
Lack of fit	0.6647	3	0.2216	6.16	0.0557
Pure error	0.1439	4	0.0360		
Cor total	61.31	12			
R^2=0.9868					

* X_1: extraction pressure; X_2: temperature

$p \geq 0.05$ not significant.

Table S2. Analysis of variance (ANOVA) for the response surface quadratic models for selected terpenes.

Source	Sum of squares	df	Mean square	F-value	*p*-value*
			α-Pinene		
Model	51.80	5	10.36	9.47	0.0051
X_1	30.64	1	30.64	28.00	0.0011
X_2	0.9380	1	0.9380	0.8573	0.3853
X_1^2	1.65	1	1.65	1.51	0.2589
X_2^2	18.45	1	18.45	16.87	0.0045
X_1X_2	0.8101	1	0.8101	0.7405	0.4180
Residual	7.66	7	1.09		
Lack of fit	6.12	3	2.04	5.32	0.0701
Pure error	1.54	4	0.3839		
Cor total	59.46	12			
R^2=0.8712					
			β-Pinene		
Model	22.95	5	4.59	18.42	0.0007
X_1	15.44	1	15.44	61.96	0.0001
X_2	0.1507	1	0.1507	0.6045	0.4623
X_1^2	0.3782	1	0.3782	1.52	0.2578
X_2^2	6.94	1	6.94	27.86	0.0012
X_1X_2	0.0222	1	0.0222	0.0891	0.7740
Residual	1.74	7	0.2492		
Lack of fit	1.25	3	0.4178	3.40	0.1338
Pure error	0.4911	4	0.1228		
Cor total	24.70	12			
R^2=0.9294					
			β-Myrcene		
Model	220.57	5	44.11	4.27	0.0421
X_1	110.96	1	110.96	10.75	0.0135
X_2	0.2220	1	0.2220	0.0215	0.8875
X_1^2	22.37	1	22.37	2.17	0.1845
X_2^2	87.01	1	87.01	8.43	0.0229
X_1X_2	1.50	1	1.50	0.1452	0.7145
Residual	72.27	7	10.32		
Lack of fit	54.63	3	18.21	4.13	0.1022
Pure error	17.64	4	4.41		
Cor total	292.84	12			
R^2=0.7532					
			Limonene		
Model	16.12	5	3.22	19.88	0.0005
X_1	12.02	1	12.02	74.12	< 0.0001
X_2	0.0438	1	0.0438	0.2701	0.6193
X_1^2	0.4160	1	0.4160	2.56	0.1533
X_2^2	3.59	1	3.59	22.13	0.0022
X_1X_2	0.0005	1	0.0005	0.0030	0.9578
Residual	1.14	7	0.1622		

Lack of fit	0.4926	3	0.1642	1.02	0.4716
Pure error	0.6429	4	0.1607		
Cor total	17.26	12			
R^2=0.9342					
β -Caryophyllene					
Model	41.60	5	8.32	6.02	0.0178
X_1	6.11	1	6.11	4.42	0.0736
X_2	5.98	1	5.98	4.33	0.0759
X_1^2	13.88	1	13.88	10.04	0.0157
X_2^2	15.62	1	15.62	11.31	0.0120
X_1X_2	0.1693	1	0.1693	0.1226	0.7366
Residual	9.67	7	1.38		
Lack of fit	6.56	3	2.19	2.81	0.1721
Pure error	3.11	4	0.7785		
Cor total	51.27	12			
R^2=0.8114					
Guaiol					
Model	14.69	5	2.94	8.57	0.0068
X_1	11.16	1	11.16	32.55	0.0007
X_2	0.0197	1	0.0197	0.0576	0.8172
X_1^2	0.1190	1	0.1190	0.3472	0.5742
X_2^2	2.82	1	2.82	8.22	0.0241
X_1X_2	0.9472	1	0.9472	2.76	0.1404
Residual	2.40	7	0.3428		
Lack of fit	1.96	3	0.6547	6.01	0.0579
Pure error	0.4355	4	0.1089		
Cor total	17.09	12			
R^2=0.8596					
γ-Eudesmol					
Model	15.69	5	3.14	6.29	0.0159
X_1	12.08	1	12.08	24.23	0.0017
X_2	0.0676	1	0.0676	0.1356	0.7236
X_1^2	0.1332	1	0.1332	0.2671	0.6212
X_2^2	2.59	1	2.59	5.19	0.0567
X_1X_2	1.23	1	1.23	2.46	0.1606
Residual	3.49	7	0.4987		
Lack of fit	2.85	3	0.9499	5.93	0.0592
Pure error	0.6411	4	0.1603		
Cor total	19.18	12			
R^2=0.8180					
Bulnesol					
Model	10.49	5	2.10	5.76	0.0201
X_1	9.15	1	9.15	25.11	0.0015
X_2	0.0067	1	0.0067	0.0184	0.8959
X_1^2	0.1296	1	0.1296	0.3556	0.5697
X_2^2	0.7073	1	0.7073	1.94	0.2062
X_1X_2	0.6530	1	0.6530	1.79	0.2225

Residual	2.55	7	0.3644		
Lack of fit	2.05	3	0.6836	5.47	0.0672
Pure error	0.5001	4	0.1250		
Cor total	13.04	12			
R^2=0.8044					

[*] X_1: extraction pressure; X_2: temperature

$p \geq 0.05$ not significant.

Table S3. Analysis of variance (ANOVA) for the response surface quadratic models for CBD and CBDA.

Source	Sum of squares	df	Mean square	F-value	*p*-value*
			CBD		
Model	27.07	5	5.41	17.00	0.0009
X_1	2.48	1	2.48	7.79	0.0269
X_2	10.66	1	10.66	33.48	0.0007
X_1^2	0.3306	1	0.3306	1.04	0.3422
X_2^2	12.36	1	12.36	38.81	0.0004
X_1X_2	0.4159	1	0.4159	1.31	0.2907
Residual	2.23	7	0.3185		
Lack of fit	1.68	3	0.5609	4.10	0.1031
Pure error	0.5467	4	0.1367		
Cor total	29.30	12			
R^2=0.9239					
			CBDA		
Model	762.17	5	152.43	8.37	0.0072
X_1	648.40	1	648.40	35.59	0.0006
X_2	18.66	1	18.66	1.02	0.3452
X_1^2	4.75	1	4.75	0.2609	0.6252
X_2^2	66.76	1	66.76	3.66	0.0971
X_1X_2	34.61	1	34.61	1.90	0.2106
Residual	127.52	7	18.22		
Lack of fit	80.55	3	26.85	2.29	0.2206
Pure error	46.97	4	11.74		
Cor total	889.69	12			
R^2=0.8567					

Table S4. Analysis of variance (ANOVA) for the response surface quadratic models for antioxidant and antibacterial activity.

Source	Sum of squares	df	Mean square	F-value	*p*-value*
			DPPH		
Model	134.67	5	26.93	5.93	0.0185
X_1	81.11	1	81.11	17.87	0.0039
X_2	0.6904	1	0.6904	0.1521	0.7081
X_1^2	0.6241	1	0.6241	0.1375	0.7217
X_2^2	51.99	1	51.99	11.45	0.0117
X_1X_2	2.09	1	2.09	0.4616	0.5187
Residual	31.77	7	4.54		
Lack of fit	25.80	3	8.60	5.77	0.0619
Pure error	5.97	4	1.49		
Cor total	166.44	12			
R^2=0.8091					
			MIC (B. subtilis)		
Model	620.02	5	124.00	5.05	0.0280
X_1	188.85	1	188.85	7.70	0.0275
X_2	209.24	1	209.24	8.53	0.0223
X_1^2	68.56	1	68.56	2.79	0.1385
X_2^2	4.25	1	4.25	0.1732	0.6898
X_1X_2	140.15	1	140.15	5.71	0.0482
Residual	171.75	7	24.54		
Lack of fit	31.65	3	10.55	0.3012	0.8241
Pure error	140.10	4	35.03		
Cor total	791.78	12			
R^2=0.7831					

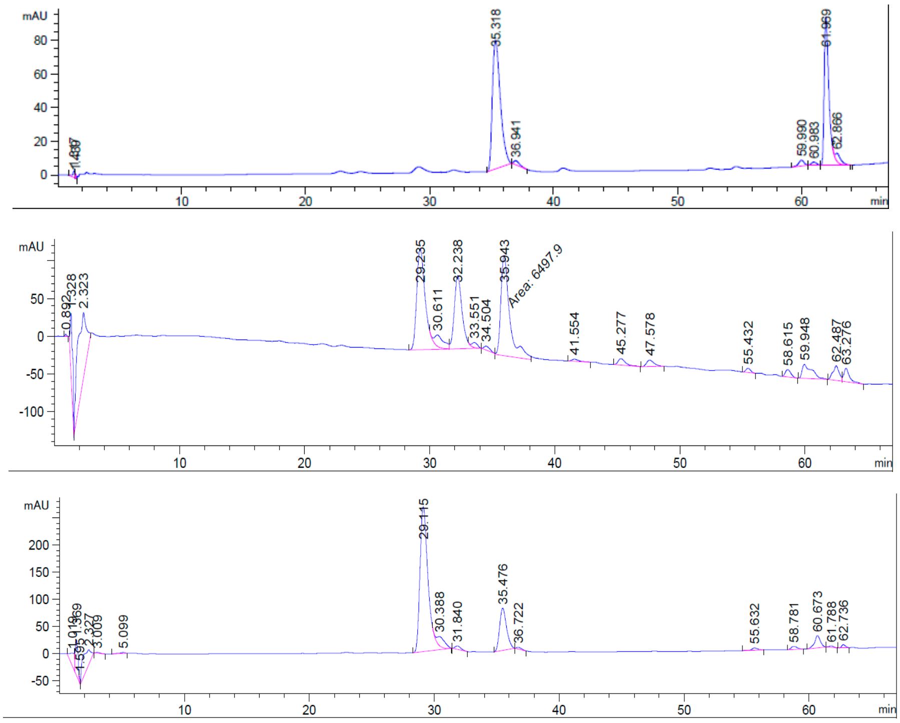

Figure S1. Representative chromatogram of the HPLC analysis of the extracts number 4; upper chromatogram at 250 nm – CBCA at 62.735 min; mean chromatogram at 210 nm – CBG at 33.243 min, THC at 47.578 min; lower chromatogram at 230 nm – CBD at 31.840 min, CBDA at 35.476 min, CBC at 55.632 min and THCA at 60.673 min.
